# Supplementary material for: Neuroinductive properties of mGDNF depend on the producer, E. Coli or human cells
Source: PLoS One. 2021 Oct 11;16(10):e0258289. doi: 10.1371/journal.pone.0258289 (PMC8504721; doi:10.1371/journal.pone.0258289)
Supplement: S1 Raw images — (PDF) [file pone.0258289.s002.pdf]

Fig1

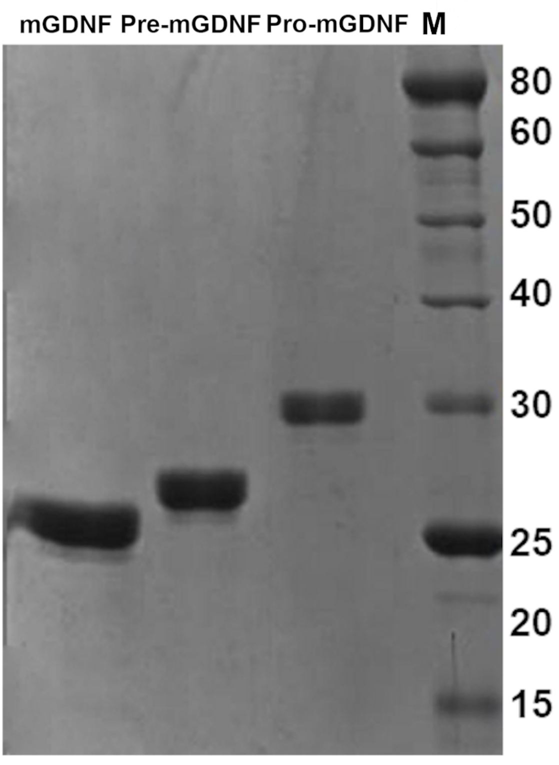

Fig2

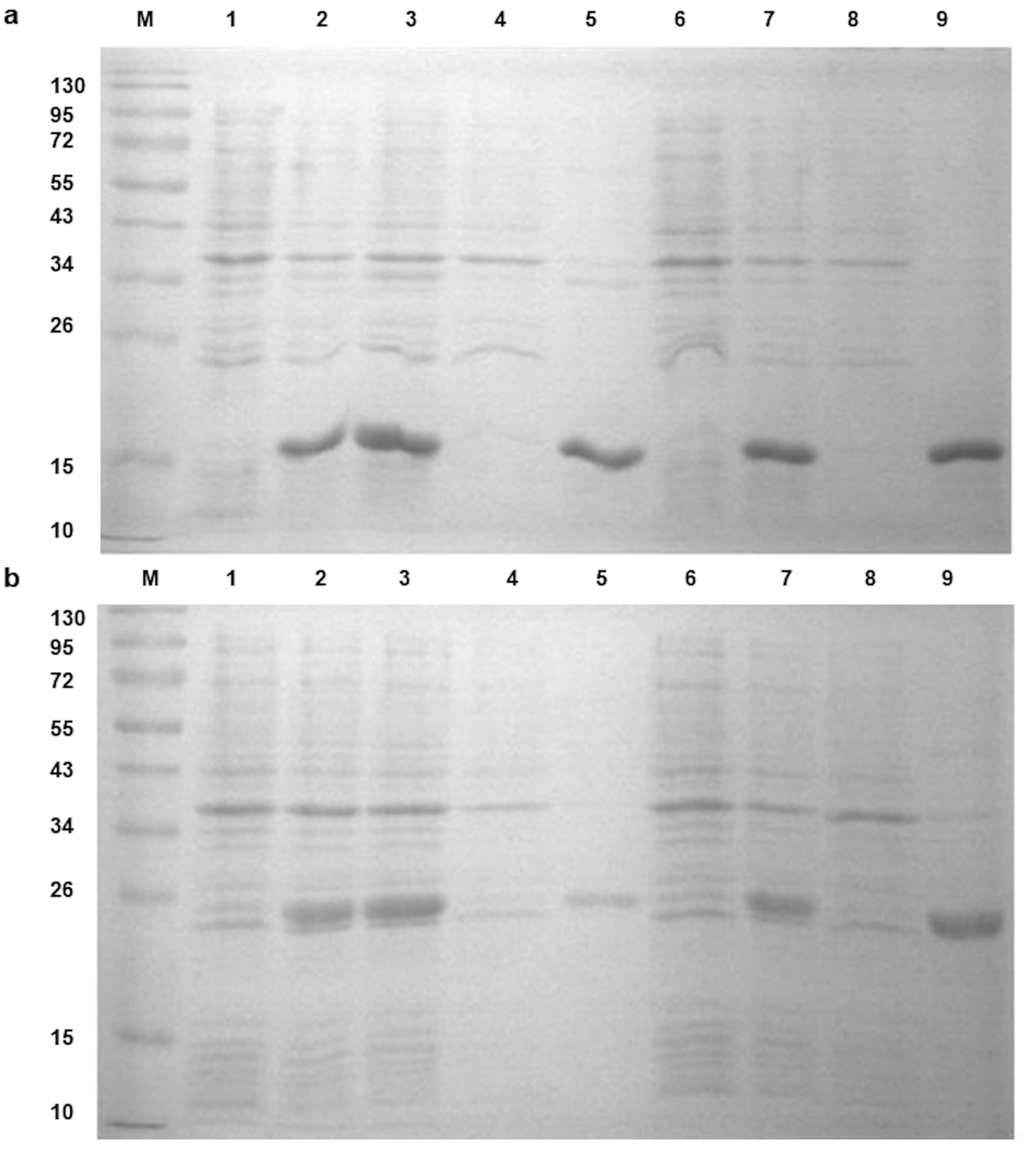

Fig2. (a) and (b): M, calibration standard; 1, prior to induction at 25°C; 2, induction after 17-h incubation at 25°C; 3, induction after 24-h incubation at 25°C; 4, supernatant after incubation at 25°C; 5, inclusion bodies after incubation at 25°C; 6, prior to induction at 37°C; 7, induction after 3-h incubation at 37°C; 8, supernatant after incubation at 37°C; 9, inclusion bodies after incubation at 37°C.

S1 Fig

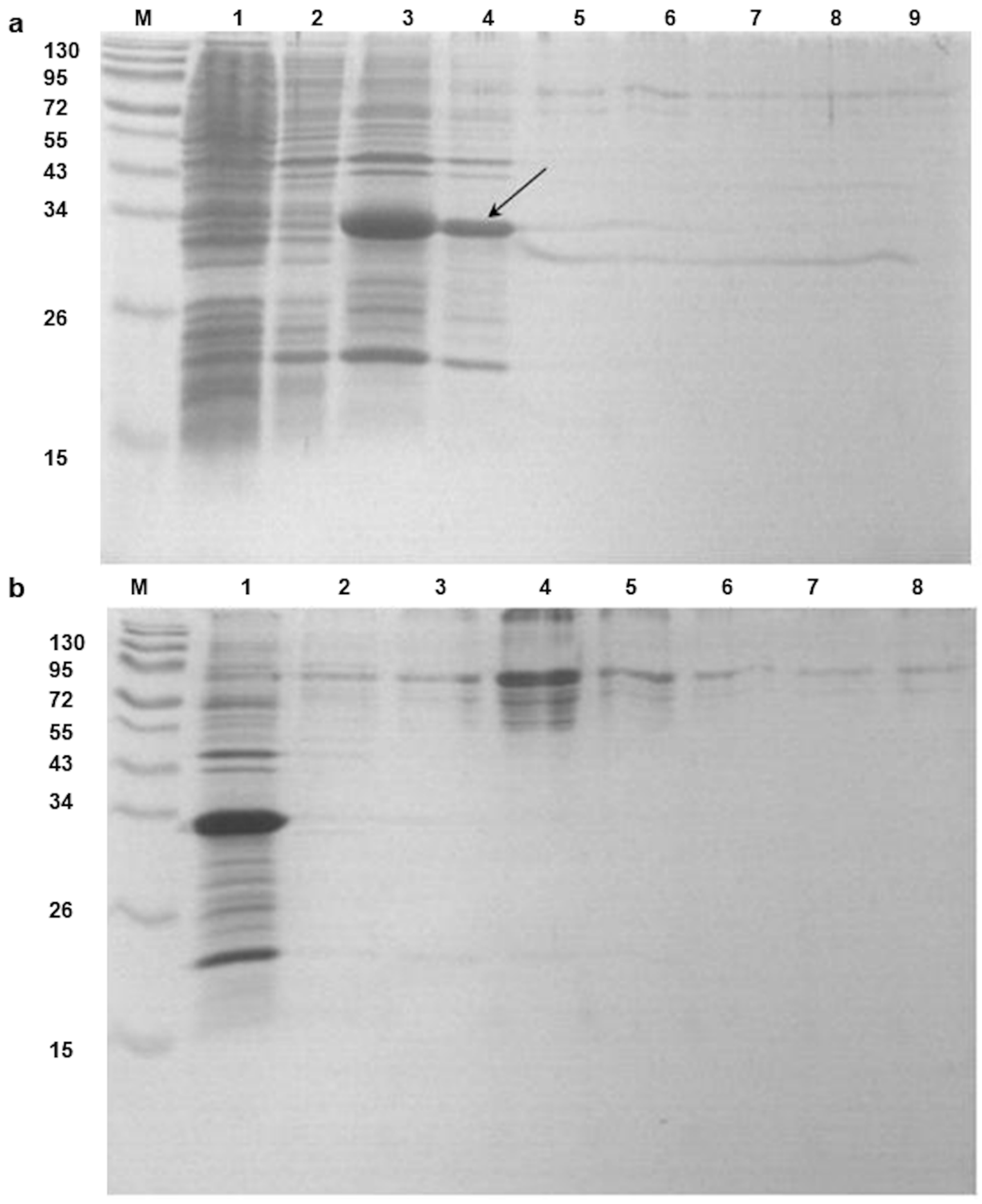

S1 Fig. (a) 1, molecular weight markers, kDa; 2, extraction stage 1 (supernatant); 3, extraction stage 2 (supernatant); 4, solubilization stage (supernatant prior to dialysis); 5, dialysate prior to filtration; 6, dialysate after filtration (0.45 μm) prior to column; 7, flow through; 8, eluate fraction 1; 9, eluate fraction 2; 10, dialysate of the fraction 1 eluate.
(b) M, molecular weight markers, kDa; 1, dialysate prior to column (supernatant without filtration); 2, flow-through; 3, eluate fraction 1; 4, eluate fraction 1 after dialysis; 5, eluate fraction 1 after dialysis and centrifugation (14,000 rpm, 10 min, 4°C); 6, eluate fraction 2; 7, eluate fraction 2 after dialysis; 8, eluate fraction 2 after dialysis and centrifugation (14,000 rpm, 10 min, 4°C).

Figs 1,2 and S1 Fig were captured by Canon i-SENSYS MF4330d.
